# Lupus Nephritis in Children: Novel Perspectives

**DOI:** 10.3390/medicina59101841

**Published:** 2023-10-16

**Authors:** Marco Pennesi, Simone Benvenuto

**Affiliations:** 1 Institute for Maternal and Child Health IRCCS Burlo Garofolo, 34137 Trieste, Italy; 2 Department of Medicine, Surgery, and Health Sciences, University of Trieste, 34127 Trieste, Italy

**Keywords:** systemic lupus erythematosus, lupus nephritis, pediatric, activity and chronicity index, voclosporin, belimumab, obinutuzumab, low disease activity, urinary CD163

## Abstract

Childhood-onset systemic lupus erythematosus is an inflammatory and autoimmune condition characterized by heterogeneous multisystem involvement and a chronic course with unpredictable flares. Kidney involvement, commonly called lupus nephritis, mainly presents with immune complex-mediated glomerulonephritis and is more frequent and severe in adults. Despite a considerable improvement in long-term renal prognosis, children and adolescents with lupus nephritis still experience significant morbidity and mortality. Moreover, current literature often lacks pediatric-specific data, leading clinicians to rely exclusively on adult therapeutic approaches. This review aims to describe pediatric lupus nephritis and provide an overview of the novel perspectives on the pathogenetic mechanisms, histopathological classification, therapeutic approach, novel biomarkers, and follow-up targets in children and adolescents with lupus nephritis.

## 1. Introduction

Systemic lupus erythematosus (SLE) is an inflammatory and autoimmune condition characterized by heterogeneous multisystem involvement and a chronic course with unpredictable flares. Despite potentially affecting every organ system, skin, joints, the central nervous system, blood cells, and kidney involvement are the most common [1]. Childhood-onset systemic lupus erythematosus (cSLE), i.e., occurring before 18 years of age, accounts for 10–20% of cases and is known for being more severe than adult SLE, both in terms of aggressive onset and disease flares [2,3].

Kidney involvement mainly presents with immune complex-mediated glomerulonephritis, commonly named lupus nephritis (LN), and represents one of the most severe manifestations of SLE. LN is more frequent in cSLE than adult SLE, developing in 32–55% of cases but also more severe with a considerable risk of long-term kidney damage [3,4,5,6,7,8,9,10]. Since the introduction of cytotoxic agents in the 1980s, long-term renal prognosis has markedly improved, with recent studies showing 92% and 86% five- and ten-year kidney survival, respectively [11]. Still, children and adolescents with LN experience significant morbidity and 19 times higher mortality than healthy children [12], highlighting the critical need for appropriate LN management for this age.

Management of LN is rapidly evolving thanks to several advances in recent research, mainly regarding new therapies but also a better understanding of pathogenesis, an improved histopathological definition, the identification of potential biomarkers for risk stratification, and new treatment targets during follow-up. Unfortunately, most clinical trials did not include pediatric patients. As the lack of data specific to children with LN led clinicians and recommendations to rely exclusively on adult therapeutic approaches, many advocate the off-label use of novel therapies in pediatric patients in order to develop clinical experience and collect specific data, pending new clinical trials to establish the best approach for pediatric LN [13].

This review describes pediatric LN and highlights novel perspectives on the pathogenetic mechanisms, histopathological classification, therapeutic approach, novel biomarkers, and follow-up targets in children and adolescents with LN.

## 2. Epidemiology

cSLE is a rare disorder, with a reported prevalence of 1.9–25.7 per 100,000 children and an incidence of 0.3–0.9 per 100,000 per year worldwide, accounting for 10–20% of SLE cases [5,14,15]. It rarely occurs before 5 years of age, with an increasing prevalence in the second decade of life [9]. Similar to adult SLE, cSLE prevalence markedly differs based on demographic factors including sex, with girls, especially adolescents, being four to nine times more involved than boys, probably due to hormonal changes in puberty [16,17], race, since non-Caucasian individuals are more frequently affected [18], and residency, with urban areas being more common than rural areas [19].

Among patients with cSLE, the rate of those developing LN varies among different registries from 32 to 55% [3,4,5,6,7,8,9,10]. LN usually develops early in the disease course, with data from the Childhood Arthritis and Rheumatology Research Alliance (CARRA) registry revealing its occurrence in 88% of cases in the first year after cSLE diagnosis and in 93% within two calendar years [7]. Remarkably, these data resulted in higher levels than previously reported in the same North American population [9], possibly reflecting an improvement in the diagnosis of LN since lower thresholds for performing kidney biopsy were recommended.

## 3. Pathogenesis

Significant progress has recently been made in understanding SLE, mainly regarding novel genetic variants and factors related to gene expression and epigenetics involved in monogenic and sporadic SLE forms. Indeed, SLE is a complex disorder resulting from genetic susceptibility and environmental exposure to factors that induce epigenetic alterations, such as sunlight, hormonal abnormalities, medications, viral infections, and air pollutants [20,21,22,23]. Among viral triggers, Epstein–Barr virus (EBV) can impair innate and adaptative immune responses but also induce plasmacytoid dendritic cells to produce type I interferon (IFN-I) and has been associated with the development of SLE in epidemiological studies [24,25]. Regarding the female predominance of SLE, it is still poorly understood whether it depends on hormonal or genetic factors. However, recent studies have focused on epigenetically-induced alterations in X-linked immunity gene expression [26]. For example, the dysregulation of XIST, a long non-coding RNA that plays a role in chromosome X inactivation and regulation of X-linked immune genes such as toll-like receptors (TLR) 7, was found in female SLE patients [27].

The role of genetic predisposition has long been recognized, with monozygotic twins showing a 10-fold increased concordance compared to dizygotic twins [28]. Currently, the risk of developing SLE is either attributed to common variants with low penetrance, rare mutations with substantial disease risk, or a combination of both [29,30]. SLE is mostly a polygenic disease, with approximately 180 susceptibility loci identified in genome-wide association studies, mainly impacting signaling involved in IFN-I and NF-kB pathways and T-cell and B-cell activation and differentiation. However, most of these variants reside in non-coding regions, making it difficult to interpret their functional implications [31]. Interestingly, recent analyses revealed that risk variants often tag a so-called super enhancer, which increases the transcription of several genes promoting broad immune activation [32].

On the other hand, monogenic forms of cSLE are rarely observed. They are based upon single gene defects involving several immune pathways, such as IFN-I, nucleic acid sensing, repair or degradation, complement system, phagocyte oxidase system, apoptosis, and B-cell development [33,34]. In particular, deficiencies in the serum nuclease DNASE1, DNASE1L3, and intracellular DNASE2 are listed among the defects of DNA degradation [35,36,37]. Complement defects include mutations in the C1q gene with about 90% penetrance and severe disease course, C2 deficiency, which is the most common, or C4 deficiency [38,39,40]. Genetic analysis is mandatory for cSLE cases with a very early onset.

Recent studies investigated epigenetic modifications to chromatin in SLE patients, identifying hypomethylation of several IFN-I-regulated gene loci [41]. Among those, absent in melanoma 2 (AIM2) is involved in the sensing of cytosolic dsDNA; sorting nexin 18 (SNX18) mediates autophagosome formation in neutrophils. At the same time, demethylation of the N-acetylgalactosaminyltransferase 18 (GALNT18) gene is associated with active LN [42,43,44]. IL-6 and TREML4 hypomethylation, the latter being involved in TLR7 signaling, are also reported [45,46]. 

Whatever the triggering mechanism, subsequent events rely on complex immune dysregulation involving innate and adaptive immunity. Loss of immune tolerance towards self-antigens, which persist long enough to stimulate an immune response because of impaired clearance of dead cells and genetic material, is a crucial step in SLE pathogenesis [47]. Dendritic cells, acting as the primary antigen-presenting cells (APC), play a significant role in innate immune system activation. On the one hand, they express TLR-7 and -9, which act as RNA and DNA antigen sensors and represent a target for antimalarial drugs [48]. Their activation involves myeloid differentiation response gene-88 (Myd-88) and IL-1 receptor-associated kinase 4 (IRAK4) to activate interferon regulatory factor 3 (IRF3) and IRF7, with IFN-I production [49]. Dendritic cells directly produce type I IFN, which drives inflammation to activate different cell types, particularly B-cells [50]. Neutrophils also play a role in SLE pathogenesis at several levels. They can produce IFN-I independently of TLR stimulation, induce abnormal B-cell development, and represent a source of self-antigens in impaired phagocytic capacity [51]. Low-density granulocytes (LDG), a subtype of neutrophils, activate CD4+ T-cells and may form neutrophil extracellular traps (NETs), which damage endothelial cells and are elevated in patients with LN [52,53]. Monocytes and macrophages contribute to the secretion of many soluble products, including tumor necrosis factor (TNF), IL-1, IL-6, IL-10, IL-12, and B lymphocyte stimulator (BLyS). Recently, a role in SLE pathogenesis was revealed for IL-18, whose blood levels were more elevated in patients with higher disease activity [54]. Finally, complement system relevance has also been recognized, and its deficiency has been associated with more severe forms of cSLE [39,55].

Interestingly, while studies in adults failed to demonstrate a role for the IFN signature in classifying patients or assessing disease activity, a recent study found that cSLE patients with normocomplementemia had higher IFN scores and lower disease severity than patients with hypocomplementemia, which conversely showed a higher titer of anti-dsDNA antibodies. A novel disease model was therefore proposed, at least for cSLE, with two distinct subsets of disease where the high IFN, normocomplementemic, and possibly milder phenotype group of children sets closer to autoinflammation in the pathogenic continuum with autoimmunity, which has usually been considered prevalent in SLE patients [56]. Identifying this subgroup may help in patients’ stratification in new clinical trials and support the investigation of drugs targeting the IFN pathway [57].

Adaptative immunity is also involved in SLE pathogenesis, with T-cell and/or B-cell functional or phenotypic anomalies potentially inducing loss of immune tolerance and contributing to a pro-inflammatory status. On the one hand, impaired production of IL-2 favors effector T-cells such as the T helper 17 (Th17) phenotype, reducing regulatory T-cells [58]; immunosuppressive agents such as mycophenolate mofetil (MMF) and azathioprine (AZA) interfere with T-cell function. Th17 cells are the primary source of IL-17, which recruits neutrophils and B-cells, further activates innate immunity, and exacerbates tissue injury [59]. CD8+ T-cells are gaining interest given their identification in the kidney interstitium, suggesting a role in LN pathogenesis [60]. T follicular helper (Tfh) cells may be among the most active to induce B-cell differentiation [61]. On the other hand, high levels of B lymphocyte stimulator (BLyS) usually characterize cSLE [62], which is responsible for autoantibody production, the hallmark of SLE. In this field, treatments targeting the B-cell pathway, such as rituximab (RTX) and belimumab, are emerging. While rapidly expanding plasmablasts represent a source of anti-dsDNA autoantibodies and express CD19, being a potential target for anti-CD19 chimeric antigen receptor (CAR) T cell therapy, long-lived plasma cells are the primary source of anti-extractable nuclear antigen autoantibodies (e.g., anti-Sm and anti-RNP) and do not express CD19. Therapies targeting CD38 or BCMA, both expressed by the latter, may overcome this limitation. Finally, age-associated B cells (ABCs) represent an emerging subset of B-cells whose proliferation and autoantibody production are driven by the TLR7 pathway and not by the B-cell receptor [63]. Characterizing their cell surface features may offer new therapeutic targets.

**Figure 1:**
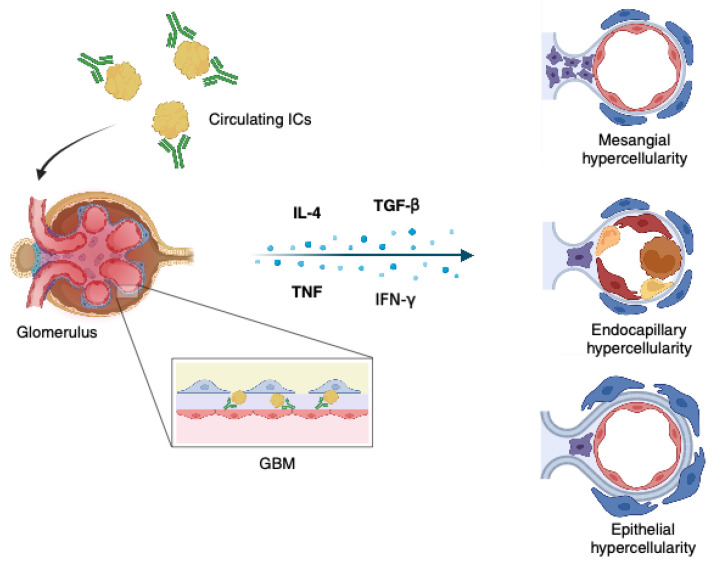
Pathogenesis of lupus nephritis. ICs: immune complexes; GBM: glomerular basement membrane; IL-4: interleukin-4; TGF-β: transforming growth factor-beta; TNF: tumor necrosis factor; IFN-γ: interferon-gamma. Created with BioRender.com.

Large amounts of autoantibodies may finally react with self-antigens to form circulating immune complexes (ICs), with a deposit in various tissues, particularly the glomerulus, representing the first insult to determine the development of LN. The subsequent activation of classical complement, macrophage, and neutrophil pathways triggers the production of proinflammatory and profibrotic cytokines, such as interleukin-4 (IL-4), transforming growth factor-beta (TGF-β), TNF, and interferon-gamma (IFN-γ), leading to podocyte injury, mesangial, endocapillary, and epithelial hypercellularity, extracellular matrix deposition, and eventually renal impairment [64,65,66]. Thrombotic microangiopathy (TMA), IC-mediated tubulointerstitial nephritis, and amyloidosis represent other renal disorders associated with SLE apart from LN [67]. Pathogenetic mechanisms underlying LN are shown in Figure 1.

## 4. Pathology

The LN histopathological classification is based on the recommendations from the International Society of Nephrology and the Renal Pathology Society (ISN/RPS), with their original version released in 2003 still being the most used in LN clinical trials [68]. A kidney biopsy, including, as a general rule, a minimum of 10 glomeruli, should be evaluated by a renal pathologist experienced in LN [69].

The ISN/RPS classification system recognizes six classes of nephritis, which carry a prognostic value as they are associated with disease severity and long-term renal outcome [70]. LN classes I and II are characterized by immune complex deposition in mesangial cells, with various degrees of mesangial proliferation. However, this expansion does not usually determine proliferative or sclerosing glomerular injury, thanks to mesangial cells’ high regenerative capacity, so these classes are associated with a good prognosis [65]. While isolated detection of IC deposition identifies class I, class II is further characterized by mesangial hypercellularity or mesangial matrix expansion. Subendothelial IC deposition with endocapillary hypercellularity, alone or in combination with mesangial IC deposition, is the hallmark of LN classes III and IV, which differ based on the percentage of involved glomeruli with a cut-off value of 50%. Remarkably, severe lesions in these classes may produce crescents due to the rupture of glomerular capillaries with leakage of fibrinogen and other mitogenic proteins in the urinary space. LN class V derives instead from the subepithelial deposition of ICs, which have either crossed the glomerular basement membrane or formed locally after podocyte injury. It is often associated with nephrotic range proteinuria and may occur alone or in combination with class III or IV lesions. Finally, in class VI, over 90% of evaluated glomeruli show glomerulosclerosis, determined by glomerular, vascular, and tubulointerstitial injury. Class III and IV lesions are predominant in children with LN, accounting for up to 75% of cases, and carry the worst renal prognosis, so most clinical trials focus on these patients, sometimes including class V LN [9].

In 2018, ISN/RPS revised the previous classification system to clarify some definitions based on existing evidence and experts’ opinions while pursuing harmonization among definitions for certain lesions also encountered in renal diseases other than LN [71]. Firstly, a threshold of four cells surrounded by the matrix in the peripheral mesangial area to define mesangial hypercellularity was proposed, similar to the Oxford classification of IgA nephropathy [72]. This approach allows a precise differentiation between LN classes I and II. Many other clarifications were given regarding classes III and IV: the term “endocapillary hypercellularity” should be used instead of “proliferation”, because of the usual predominance of inflammatory cells rather than actual cell proliferation; membranoproliferative glomerulonephritis (MPGN) pattern should be included in class III and IV LN; crescents, i.e., lesions consisting of extracapillary hypercellularity, were classified in “cellular” if made of more than 75% cells and fibrin, “fibrous” if consisting of more than 75% fibrous matrix, and “fibrocellular” when of mixed nature; the term “adhesion” was preserved for areas of isolated continuity of extracellular matrix between the glomerular tuft and the capsule, making a distinction from both crescents and segmental sclerosis; the subdivision of class IV LN into “segmental” and “global” was eliminated due to low reproducibility and uncertain clinical significance [73]; fibrinoid necrosis, not previously taken into account, was considered to be noticed in class III and IV in order to assess its clinical significance in future studies. Finally, the active (A) and/or chronic (C) descriptors for class III and IV lesions were eliminated, advocating the usage, for all classes, of a modified National Institute of Health (NIH) activity and chronicity index, able to provide more information, as summarized in Table 1.

Validation studies confirmed the higher precision of the revised ISN/RPS classification system in predicting clinical outcomes compared to the 2003 classification [74,75]. However, the 25% and 50% thresholds for crescents were questioned in cSLE patients. The new proposed 10% and 25% thresholds better reflected disease activity and predicted kidney outcomes in this population [76].

Further studies are needed to investigate the clinical validity of many changes introduced by the ISN/RPS 2018 revision, such as the cutoff values for mesangial hypercellularity, the significance of MPGN pattern, adhesions, and fibrinoid necrosis, the thresholds for activity and chronicity index scores (including the double weight of fibrinoid necrosis and crescents), and to possibly improve the definitions of class V lesions, where the clinical significance of associated mesangial hypercellularity was not evaluated, and class VI, which is rarely seen and should therefore be redefined with new cutoffs or even eliminated. Finally, the role of other types of injury not currently included in classification systems should be addressed, such as vascular lesions, which currently have no standardized approach to distinguish LN-related lesions such as vasculopathy associated with IC deposition, vasculitis, and TMA, and “lupus podocytopathy”, which can be found through electron microscopy and implies humoral, toxic, infectious, or genetic factors of podocyte injury, such as the apolipoprotein L1 (APOL-1) risk alleles reaching a 30% prevalence in individuals of West African origin [77,78,79].

## 5. Diagnosis

Clinical manifestations of cSLE are very heterogeneous. Extensive cohort studies proved that fever, hepatomegaly, and splenomegaly were more frequent in younger children compared to photosensitivity, weight loss, and leukopenia, which were typical of adolescents [80]. In most cases, however, early diagnosis was challenging, and the median time interval from disease onset was ≥3 months [81]. As for adults, the cSLE diagnosis is supported by classification criteria. After the 1997 American College of Rheumatology (1997-ACR) criteria and the 2012 Systemic Lupus International Collaborating Clinics (2012-SLICC) criteria [82,83], in 2019, the European League Against Rheumatism (EULAR) and the ACR released a new set of classification criteria, with the introduction of antinuclear antibodies (ANA) positivity (≥1:80) as an entry criterion and seven clinical domains (constitutional, hematologic, neuropsychiatric, mucocutaneous, serosal, musculoskeletal, and renal) and three immunological (antiphospholipid antibodies, complement, and SLE-specific antibodies such as anti-dsDNA or anti-Smith antibody) to group weighted criteria used to calculate a score, suggestive for SLE if ≥10 [84]. Remarkably, histological evidence of class III or IV LN and class II or V LN carry the highest scores among these criteria, with 10 and 8 points, respectively. The 2019-EULAR/ACR criteria showed improved sensitivity (0.96) and specificity (0.93) compared to the 1997-ACR and the 2012-SLICC criteria, respectively. Their better performance was confirmed in the cSLE population, although they presented lower specificity (0.89) than adults [85]. The renal domain of the 2019-EULAR/ACR classification is shown in Table 2.

LN is more frequent in cSLE than adult SLE, developing in 32–55% of cases but also being more severe [3,4,5,6,7,8,9,10]. It often emerges as a presenting manifestation, but close surveillance is mandatory in non-renal SLE cases, particularly in the first three years after disease onset [86]. LN could present with proteinuria of varying degrees, glomerular hematuria, and/or decreased glomerular filtration due to focal or diffuse glomerular damage. Clinical presentation could, therefore, vary from microscopic hematuria and/or mild proteinuria to nephrotic syndrome, rapidly progressing glomerulonephritis, and chronic (CKD) or acute kidney injury (AKI), with the latter carrying a high long-term risk of kidney failure, particularly in the case of stage 3 dialysis-requiring AKI [87]. In some cases, tubule-interstitial damage occurs with urine concentration and electrolyte reabsorption abnormalities [88].

Clinical findings do not accurately predict renal biopsy findings, as even mild urinary abnormalities could be associated with active lesions [89,90]. All adult and pediatric SLE recommendations, therefore, state that percutaneous kidney biopsy is mandatory in SLE patients with suspected renal involvement, i.e., presenting glomerular macroscopic or microscopic hematuria or cellular cast, proteinuria > 0.5 g/24 h (or spot urine protein-to-creatinine ratio (UPCR) > 0.5 mg/mg), hypertension, and not otherwise explained decreased glomerular filtration rate (GFR) [69,91,92]. Earlier use of kidney biopsy was associated with improved prognosis [93]. However, according to the 2017 Single Hub and Access Point for Paediatric Rheumatology in Europe (SHARE) pediatric recommendations, in contrast with the adult diagnostic pathway, it is important not to misdiagnose proteinuria and exclude orthostatic or postural proteinuria first in cases of suspected cSLE, as this is the most common cause of proteinuria in adolescents [69,94].

## 6. Treatment

Large clinical trials to guide the treatment of children with LN are lacking, and most available studies report the results of adult regimens applied to the pediatric population. Based on these limited data, the CARRA and SHARE-specific recommendations for pediatric LN were developed in 2012 and 2017, respectively [69,95]. Pending pediatric trials, clinicians, therefore, often need to rely on adult studies and recommendations, namely the 2019 EULAR and European Renal Association-European Dialysis and Transplant Association (EULAR/ERA-EDTA) and the 2021 Kidney Disease: Improving Global Outcomes (KDIGO) [91,92]. However, age-specific issues such as adherence to treatment, growth, fertility, and psychosocial concerns should always be addressed when treating pediatric LN. Below is a summary of the current standard of care, followed by a discussion of the relevant literature regarding emerging therapies.

### 6.1. General Treatment

General management of cSLE patients includes sunscreen protection, a balanced diet, routine physical activity, and control of other cardiovascular risk factors such as obesity, dyslipidemia, and type 1 diabetes [96,97]. Given their increased risk of infection, which can induce disease flares and is a significant cause of mortality [98], immunization with inactivated vaccines is strongly recommended; live attenuated vaccines are usually avoided in immunosuppressed patients, although no severe events were reported in a case series of cSLE patients receiving the measles, mumps, rubella, and varicella-zoster vaccinations [99]. Adjuvant proteinuria and arterial hypertension treatment, which affect almost one-third of cSLE patients by the third year of disease [100], is also recommended for their impact on long-term prognosis. Angiotensin-converting enzyme inhibitors (ACE-I) and angiotensin receptor blockers (ARB) are the preferred renoprotective therapies [69]. Finally, mental health issues such as anxiety and depression in more than one-third of cSLE patients should induce referral to professionals such as psychiatrists or clinical psychologists [101].

### 6.2. Immunosuppressive Treatment

#### 6.2.1. Goals of Treatment

While the baseline degree of proteinuria does not predict subsequent renal function deterioration, the best predictor of long-term renal outcome is proteinuria at 12 months [11,102,103]. A complete renal response is therefore defined by proteinuria <0.5 g/24 h (or spot UPCR < 0.5 mg/mg) with normal renal function (estimated glomerular filtration rate (eGFR) > 90 mL/min/1.73 m^2^) by 12 months. A partial renal response is instead defined as a ≥50% reduction in proteinuria (at least subnephrotic) with normalized/stabilized eGFR within 6 months (or 12 months for those with nephrotic baseline proteinuria) [69,91]. Still, up to 50% of patients not reaching complete renal response show stable long-term renal function [104]. Although specific cutoff values for complete and partial renal response in pediatric LN patients still must be established, stricter control of proteinuria could likely be beneficial given the increased severity of the disease at this age.

Further therapy goals are reducing disease activity, preventing flares and chronic kidney damage, minimizing drug toxicity, and improving patient quality of life [88]. 

#### 6.2.2. Initial Treatment

All adult and pediatric guidelines recommend the antimalarial drug hydroxychloroquine (HCQ) for all cSLE patients, given the reduced risk of flares, end-stage kidney disease (ESKD), and death associated with its use [69,105,106]. The daily dose should be <5 mg/kg to avoid retinopathy. The American Academy of Ophthalmology recommends a baseline ophthalmologic evaluation followed by annual screening starting from the fifth year of HCQ therapy or yearly from baseline in cases of risk factors such as CKD grade 3 or below [107].

Without pediatric studies for class I and II LN, the SHARE initiative recommends low-dose oral glucocorticoid therapy (0.25–0.5 mg/kg/day, maximum 30 mg/day), tapered over 3–6 months. Disease-modifying antirheumatic drugs (DMARDs), such as MMF or AZA, may be added to class II LN in cases of persisting proteinuria or failing to taper glucocorticoids after 3 months, after a re-evaluation of the kidney biopsy to exclude misclassification [69]. According to KDIGO, nephrotic range proteinuria in class I or II LN patients should raise suspicion of lupus podocytopathy. Similar to minimal change disease, this condition usually responds to glucocorticoid monotherapy, requiring additional immunosuppressive agents such as MMF, AZA, RTX, or a calcineurin inhibitor (CNI) in case of relapse [92,108,109].

According to CARRA and SHARE recommendations, initial treatment for class III and IV LN should include high-dose glucocorticoids combined with either MMF (1200 mg/m^2^/day, maximum 2000 mg/day; in case of poor response, 1800 mg/m^2^/day, maximum 3000 mg/day) or intravenous (IV) cyclophosphamide. This approach is based on milestone papers demonstrating the superiority of CYC compared to high-dose prednisone alone [110], then the comparable efficacy between MMF and IV CYC [111,112], and finally, the similar efficacy but improved side-effect profile of low-dose IV CYC compared to high-dose IV CYC in the Euro-Lupus Nephritis Trial [113]. SHARE recommendations indicate either high-dose (6 monthly pulses, 500–750 mg/m^2^, maximum 1000–2000 mg/dose) or low-dose (6 pulses every 2 weeks, 500 mg/pulse) IV CYC, in case of CYC usage. While CYC is the only drug with available long-term data demonstrating its efficacy in preserving kidney function [114,115], its well-known toxicity profile, including delayed events such as hematologic malignancies (in case of total lifetime exposure >36 g), myelofibrosis (>80 g), and infertility (>7.5–15 g/m^2^ for pediatric patients, >300 mg/kg for adults), together with the observations that effective induction of renal response is associated with favorable long-term kidney outcomes [116,117], favors MMF use, especially in the pediatric population. In cases of intolerance to MMF (i.e., adverse gastrointestinal effects), other mycophenolic acid analogues (MPAAs) may be introduced [118]. Low-dose IV CYC proved to have a better safety profile than high-dose and should therefore be considered in patients suspected of non-compliance with oral medication [69,119]. Oral CYC (1–1.5 mg/kg/day, maximum 150 mg/day) is equally effective but more toxic than high-dose IV CYC. However, the studied oral treatment dose and duration were higher and longer than those currently recommended [120].

The best initial glucocorticoid dosing regimen needs to be adequately studied. While IV methylprednisolone pulses (3 daily doses of 250–1000 mg each) are commonly used in patients with acute and severe reduction in kidney function, nephrotic range proteinuria, a high proportion of crescents, and/or a vascular lesion on kidney biopsy, recent clinical trials indicate a general trend towards the use of glucocorticoid pulses, followed by a lower oral starting dose and/or a more rapid taper of oral glucocorticoid [92,121,122]. Avoiding excessive steroid exposure is even more relevant in children, given the implications for growth, drug adherence, and psychosocial issues [123,124]. Examples of tapering regimens based on recent trials are shown in Table 3. 

Finally, the SHARE initiative recommends low-dose oral glucocorticoid therapy (0.5 mg/kg/day) combined with MMF for pure class V LN [69]. Alternative options include CNI, RTX, or IV CYC [125,126,127].

#### 6.2.3. Subsequent Treatment

Maintenance treatment is commonly based on either MMF or AZA in adults and children. Given the conflicting results of studies evaluating these two drugs in pediatric LN [128,129,130,131,132], SHARE did not recommend a specific one. However, two adult studies directly comparing MMF and AZA, namely the MAINTAIN and the ALMS trials, demonstrated more adverse effects for AZA, which in the latter was also associated with an increased rate of LN flares and other treatment failure endpoints such as requirement for rescue therapy, persisting impaired renal function, ESKD, and death [133,134]. EULAR/ERA-EDTA and KDIGO recommend MMF as the first-choice agent for the maintenance phase [91,92]. Alternative options, mainly supported by studies in the Asian population, include CNIs, mizoribine (100–200 mg twice or three times per day), and, based on a recent trial, leflunomide (20 mg/day) [135,136,137].

Therapy de-escalation could be contemplated in patients achieving a complete renal response without ongoing extrarenal manifestations. Glucocorticoids, whose daily dose should be ≤7.5 mg prednisone (or equivalent) during the maintenance phase, should be tapered first. Early discontinuation of glucocorticoids revealed a significantly increased flare rate in a recent trial [138], while its replacement with RTX showed promising results, although limited to a cohort [139]. Both SHARE and KDIGO recommend a total duration of immunosuppression (initial phase plus maintenance) of at least 3 years, while EULAR/ERA-EDTA advises 5 to 6 years based on the time of occurrence of most kidney flares in European cohorts [103,140,141]. A recent trial failed to demonstrate the non-inferiority of maintenance therapy discontinuation after 2–3 years compared to its continuation for 2 more years [142]. Clinical trials comparing flare rates between these two approaches in pediatric LN are warranted.

#### 6.2.4. Refractory Disease and Relapse

Defining refractory LN is often difficult due to the heterogeneous response trajectory of each patient. Although most clinical trials evaluate response at 6–12 months, according to the post hoc analysis of the ALMS trial, a 2-month time frame could be sufficient to evaluate improvement [143]. On the other hand, patients without a complete or partial renal response may not need any therapeutic change if they show slow but continuous improvement.

As the prevalence of non-adherence in SLE patients could be >60% [144], assessing compliance with treatment, including drug level measurement, is the recommended first step in cases of refractory disease. All guidelines recommend switching between first-line agents, including the possibility of using CNIs combined with glucocorticoids and MMF [145,146,147,148], or RTX, supported by observational studies in adult and pediatric LN [149,150,151,152].

All LN clinical trials investigating induction therapies include patients with flares. LN relapses are generally treated with the same initial therapies used for the disease onset. However, patient preference or tolerance, cumulative exposure to CYC, and disease activity should be carefully evaluated. Interestingly, some studies suggest that LN flares may be preventable in some patients by increasing their immunosuppression based on increased anti-dsDNA and C3 levels [153,154,155].

**Figure 2:**
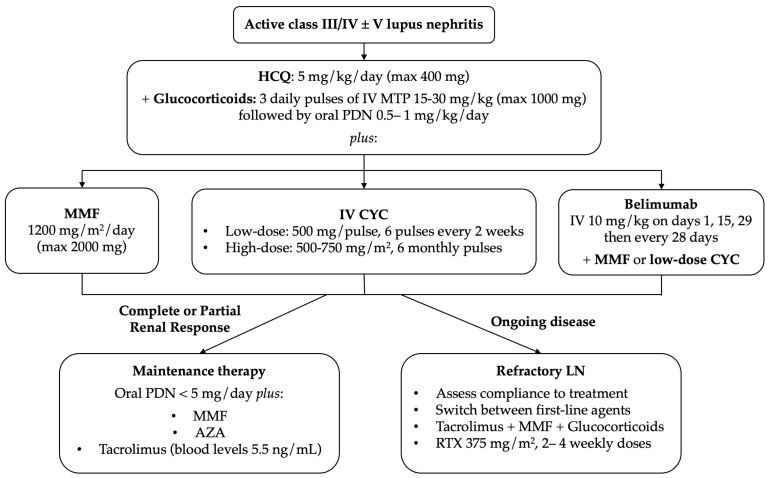
Therapeutic approach to active class III/IV alone or combined with class V LN. HCQ: hydroxychloroquine; IV: intravenous; MTP: methylprednisolone; PDN: prednisone; MMF: mycophenolate mofetil; CYC: cyclophosphamide; AZA: azathioprine; LN: lupus nephritis; RTX: rituximab.

The therapeutic approach to active class III/IV alone or combined with class V LN is summarized in Figure 2.

### 6.3. Emerging Therapies

Current treatment regimens have produced a substantial improvement in renal outcomes. However, up to 45% of patients do not achieve remission at 6 months [112]. These data highlight the urgent need for new therapies for LN. Clinical trials explored the efficacy of treatments addressing different immune targets, showing no benefit or inferiority to standard-of-care for most of these therapies, such as plasmapheresis [156], the anti-IL-6 antibody sirukumab [157], the T-cell co-stimulation inhibitor (CTLA4-Ig) abatacept [158,159], DMARDs such as AZA and leflunomide [160,161,162], and anti-CD20 such as rituximab and ocrelizumab [163,164,165].

In recent years, CNIs such as tacrolimus and cyclosporine, which act as T-cell activation inhibitors and podocyte cytoskeleton modulators, have received increased consideration for LN initial therapy, mainly due to the results of a few trials performed in the Asian population. In the largest trial, performed in Chinese patients with serum creatinine ≤ 3.0 mg/dL, a triple regimen including glucocorticoids, low-dose MMF (1 g/day), and tacrolimus (4 mg/day, with levels of 5.2–5.5 ng/mL) was compared to the standard glucocorticoids plus high-dose IV CYC regimen, showing earlier achievement of renal response. However, the renal response rate was similar after two years of treatment [166]. Another study performed in Japan demonstrated a high early response rate for a triple immunosuppressive regimen including glucocorticoids, tacrolimus, and low-dose CYC [167]. Children and adolescents with LN were included in a large meta-analysis involving 4222 patients, demonstrating that the combination of MMF and CNIs was the most effective regimen for remission induction [168]. The generalizability of these data in non-Asian populations, CNIs long-term effectiveness and safety regarding acute and chronic nephrotoxicity, optimal dose, and duration of treatment remain to be established. Nevertheless, as suggested by KDIGO recommendations, the triple regimen (CNI, MMF, and glucocorticoids) may also be considered a second-line treatment for LN initial therapy in children and adolescents [92].

A new CNI was included among initial therapy regimens by KDIGO and approved in 2021 by the Food and Drug Administration (FDA) for active LN adult patients, namely voclosporin. This cyclosporin analogue shows improved pharmacokinetic-pharmacodynamic correlation, not requiring drug-level monitoring. Its approval was based on a phase II (AURA-LV) and a phase III (AURORA 1) clinical trial, both including adult patients of various ancestry with eGFR ≥45 mL/min/1.73 m^2^, comparing voclosporin (23.7 mg twice per day) or placebo in addition to standard MMF and glucocorticoid initial therapy [121,169]. Renal response rate at 1 year was almost doubled in the voclosporin group (44% versus 23%) with similar adverse events [170]. Further studies are needed to confirm the generalizability of these results in pediatric patients concerning both the effectiveness of voclosporin and its potential chronic nephrotoxicity. Remarkably, the AURORA 2 extension study assessing voclosporin efficacy and safety in adult patients after 3 years of treatment confirmed the improved renal response rate compared to placebo (51% versus 39%), proving stable eGFR in both treatment groups [171].

B-cell targeting is gaining new interest after the negative results obtained with rituximab and ocrelizumab, possibly due to incomplete B-cell depletion [172,173], thanks to two new agents, namely belimumab and obinutuzumab. Belimumab is a monoclonal antibody blocking the soluble factor BLyS, approved by the FDA for extra-renal manifestations of adult and pediatric (>5 years of age) SLE based on two large phase III adult trials (BLISS-52 and BLISS-76) and a phase II pediatric trial (PLUTO Part A) [174,175,176]. While these trials excluded patients with severe active LN, a post hoc analysis indicated better renal outcomes in the belimumab arms [177]. The subsequent BLISS-LN trial randomizing adult active LN patients to receive belimumab (10 mg/kg IV on days 1, 15, 29, then every 28 days) or placebo in addition to standard therapy (MMF or low-dose CYC plus glucocorticoids) proved higher renal response rates at 2 years for belimumab-treated patients, and especially those receiving MMF, with an excellent safety profile [178]. In 2020, the FDA approved belimumab for adults with active LN, and these results led to the consideration of this drug by KDIGO and EULAR/ERA-EDTA recommendations [91,92]. Finally, in 2022, the FDA also approved this drug for pediatric LN, although evidence of its effectiveness in this population is still very limited [179].

Obinutuzumab is a new type II anti-CD20 monoclonal antibody, revealing higher antibody-dependent cellular cytotoxicity (ADCC) and direct B-cell killing than rituximab. In the phase II trial NOBILITY, when added to MMF and glucocorticoids and compared to placebo, obinutuzumab (1000 mg on day 1 and weeks 2, 24, and 26) showed increased complete renal response rates both at 1 year (35% versus 23%) and 2 years (41% versus 23%), with no serious adverse events [180]. A phase III trial (REGENCY) is currently ongoing.

Among B-cell targeting therapies, ofatumumab, an anti-CD20 monoclonal antibody, resulted in a safe and effective alternative in three cSLE patients allergic to rituximab [181]. At the same time, telitacicept, which inhibits both BLyS and a proliferation-inducing ligand (APRIL), led to promising results, including renal outcomes, in a small cohort of refractory cSLE patients [182]. 

Anti-IFN therapies, such as sifalimumab (anti-IFN-α monoclonal antibody) and anifrolumab (anti-type I IFN receptor monoclonal antibody), gained increasing interest after the positive results in SLE patients without LN [183,184]. In the phase II TULIP-LN trial, an intensified regimen of anifrolumab (IV 900 mg × 3 monthly, 300 mg monthly thereafter) alongside MMF and glucocorticoids directed to a higher complete renal response rate at 1 year compared to placebo (45% versus 31%), although with a more elevated incidence of herpes zoster, primarily mild or moderate and cutaneous [185]. The extension study confirmed a satisfactory safety and tolerability profile and consistent efficacy (CRR of 27% versus 18% for placebo) after the second year [186].

Janus kinase (JAK) inhibitors represent another class with positive, although limited, evidence in cSLE patients [187]. A new medication for SLE patients could be represented by iberdomide, a cereblon modulator targeting the degradation of two transcription factors, Ikaros and Aiolos, involved in leukocyte development and autoimmunity, which brought positive results in a recent 24-week phase 2 trial [188]. Finally, anti-CD19 chimeric antigen receptor (CAR) T cell therapy was tolerable and highly effective in five adolescents and young adults with refractory SLE [189].

Many other agents are currently under investigation in clinical trials, including ravulizumab and vemircopan (complement inhibitors), secukinumab and vunakizumab (anti-IL17A), and daratumumab (anti-CD38) [13]. All of these new agents should undergo a thorough investigation before pediatric use. However, they potentially carry the benefit of sparing children from the long-term side effects of glucocorticoids and cytotoxic agent treatments. Table 4 summarizes the emerging therapies for LN.

## 7. Follow-Up and Prognosis

A close follow-up is essential in LN patients in order to monitor response, drug effects, and patient education, especially in the first 2–4 months after diagnosis or flares, when visits should be scheduled every 2–4 weeks according to EULAR/ERA-EDTA recommendations [91]. One of the two standardized disease activity scores validated in the cSLE population, namely the SLE Disease Activity Index 2000 (SLEDAI-2K) and the pediatric British Isles Lupus Assessment Group (pBILAG-2004), should be used at diagnosis and every visit [190]. Spot UPCR and serum creatinine should be monitored for their long-term prognostic value. At the same time, the reappearance of glomerular hematuria or cellular casts, reduction in C3/C4, and elevation of anti-dsDNA or anti-C1q antibody titers may predict a disease flare, with the latter delivering the best performance [91,191]. However, the accuracy of these conventional biomarkers is unsatisfactory [192,193], especially considering pediatric LN patients, who cannot receive repeated kidney biopsies as easily as adult patients. New urinary biomarkers have been proposed for LN activity monitoring, such as angiostatin, ceruloplasmin, osteopontin N-half (OPN N-half), TNF-like weak inducer of apoptosis (TWEAK), and interferon-inducible protein-10 (IP-10), although they are still requiring validation in longitudinal studies including pediatric patients [194,195]. Urinary vascular cell adhesion molecule-1 (VCAM1), platelet factor-4 (PF4), and the best-performing activated leukocyte CAM (ALCAM) were recently demonstrated as potential biomarkers of kidney disease activity in a cSLE cohort [196]. Remarkably, a recent study stated the emerging role of urinary CD163 in pediatric LN. CD163 is a scavenger receptor expressed by macrophages and monocytes, whose urinary concentration is in strict association with disease activity, kidney pathology, and long-term outcomes in adult LN patients [193,197,198,199]. When evaluated in a pediatric cohort, this biomarker accurately differentiated active LN patients from active non-renal cSLE, inactive cSLE, and healthy controls, also leading to a significant correlation with SLEDAI, rSLEDAI, UPCR, C3, activity and chronicity indexes, and interstitial inflammation and fibrosis on kidney biopsy [200]. Previous studies provided promising results in cSLE patients for neutrophil gelatinase-associated lipocalin (NGAL) and urinary MCP-1, although inferior to CD163 [201,202]. Finally, six of these novel urinary biomarkers, namely NGAL, MCP-1, kidney injury molecule-1, adiponectin, haemopexin, and ceruloplasmin, were combined in the renal activity index for lupus (RAIL) to provide an accurate measure of pediatric LN activity [203]. Hence, these biomarkers could represent a noninvasive LN diagnosis and follow-up method.

According to SHARE recommendations, the pediatric version of the SLICC/ACR damage index (SDI) should be used to assess cumulative damage at least yearly [69,204]. A coordinated transition program for adolescents with LN is advocated by adult and pediatric guidelines [69,91,205].

LN in cSLE carries a high risk of organ damage. Studies from the 1990s reported that 22–50% of LN children developed ESKD [206,207]. The prognosis has improved over time, with more recent investigations indicating a 15% rate of ESKD [208], but these children continue to suffer considerable long-term morbidity and mortality. Overall, the risk of developing CKD after 1, 5, and 10 years from diagnosis is 11%, 23%, and 36%, respectively [209]. According to data from the CARRA registry, only 4% of children with initial eGFR >60 mL/min/1.73 m^2^ progress to stage 3–5 CKD. In contrast, those with a reduced initial eGFR have a good chance of recovering, but this reduction persists in 22% of cases [7]. Among those developing ESKD, after 5 years, half require a renal transplant, one-third receive it, but 22% die [5,210]. The rate of post-transplant recurrence varies broadly from 0 to 30% [211]. Children with LN on maintenance dialysis mainly die after cardiovascular events or infections [212].

Decreased proteinuria and normal serum creatinine levels at 1-year follow-up predict positive renal outcomes, together with the use of antimalarials and sustained renal remission [213,214,215,216]. Decreased eGFR, especially with serum creatinine > 1.5 mg/dl at presentation, and low C3 levels predict ESKD [207,217]. At the same time, LN classes III and IV on biopsy and hypertension at any time are independent risk factors for CKD stage 3 or higher [215].

Interestingly, lupus low disease activity (LLDAS) has recently been recognized as a valid predictor of low rates of kidney flares and damage accrual in pediatric LN [218]. LLDAS is defined as SLEDAI ≤ 4; absence of SLEDAI-based activity in major organ systems and hemolytic anemia or gastrointestinal activity; no new disease activity, either clinical or serological; physician global assessment (PGA) scale ≤ 1; prednisone dose ≤ 7.5 mg/day. Following similar results obtained in cSLE and adult SLE cohorts, this study sets LLDAS as a treatment target for children with LN [219,220].

## 8. Conclusions

Despite a considerable improvement in long-term prognosis, children and adolescents with LN experience significant morbidity and mortality. Many advances have recently been made in understanding SLE pathogenesis, defining histopathological patterns, identifying novel biomarkers and follow-up targets, and introducing new safe, tolerable, and effective treatments, potentially improving pediatric LN management despite the lack of extensive data for children and adolescents.

Normocomplementemic SLE children with high IFN signatures may represent a new subset of patients with autoinflammatory pathogenesis with potential benefit from anti-IFN therapies. The revised ISN/RPS classification system provided a clear distinction between LN classes I and II, included MPGN in class III or IV, eliminated “segmental” and “global” subdivisions for class IV and “active” and “chronic” descriptors for classes III and IV, and recommended the usage of a modified NIH activity and chronicity index. Urinary CD163, VCAM1, PF4, ALCAM, and a combination of six other urinary markers (RAIL index) emerged as potential biomarkers for LN activity. The FDA-approved belimumab for cSLE may be considered among first-line agents for active pediatric LN. Rituximab or a triple regimen including CNIs, MMF, and glucocorticoids represent second-line options for refractory LN. Voclosporin, obinutuzumab, and anifrolumab yielded promising results in adult LN trials. Excessive steroid exposure may be avoided by favoring the use of glucocorticoid pulses followed by a lower oral starting dose and/or a more rapid taper of oral glucocorticoid. Recent studies do not support the early discontinuation of maintenance therapy (i.e., after 2 years of remission). LLDAS is the preferred treatment target for children with LN.

Future studies are needed to establish child- and adolescent-specific strategies and to answer the main remaining questions, such as the pathogenetic distinction among different subsets of cSLE patients, the pediatric validation of the ISN/RPS classification system, the optimal therapy for pure class V LN, the best glucocorticoid regimen, the efficacy and safety profiles of CNIs, belimumab, obinutuzumab, and anti-IFN therapies in pediatric age, the possible role of complement inhibitors, the best treatment for LN relapses and the possibility to prevent them, and the optimal duration of therapy. 

## Figures and Tables

**Table 1:** Modified NIH activity and chronicity index for LN. Adapted from [71].

Activity Index	Definition	Score
Endocapillary hypercellularity	Endocapillary hypercellularity in <25% (1+), 25–50% (2+), or >50% (3+) of glomeruli	0–3
Neutrophils/karyorrhexis	Neutrophils and/or karyorrhexis in <25% (1+), 25–50% (2+), or >50% (3+) of glomeruli	0–3
Fibrinoid necrosis	Fibrinoid necrosis in <25% (1+), 25–50% (2+), or >50% (3+) of glomeruli	(0–3) × 2
Hyaline deposits	Wire loop lesions and/or hyaline thrombi in <25% (1+), 25–50% (2+), or >50% (3+) of glomeruli	0–3
Cellular/fibrocellular crescents	Cellular and/or fibrocellular crescents in <25% (1+), 25–50% (2+), or >50% (3+) of glomeruli	(0–3) × 2
Interstitial inflammation	Interstitial leukocytes in <25% (1+), 25–50% (2+), or >50% (3+) in the cortex	0–3
Total		0–24
**Chronicity Index**	**Definition**	**Score**
Total glomerulosclerosis score	Global and/or segmental sclerosis in <25% (1+), 25–50% (2+), or >50% (3+) of glomeruli	0–3
Fibrous crescents	Fibrous crescents in <25% (1+), 25–50% (2+), or >50% (3+) of glomeruli	0–3
Tubular atrophy	Tubular atrophy in <25% (1+), 25–50% (2+), or >50% (3+) of the cortical tubules	0–3
Interstitial fibrosis	Interstitial fibrosis in <25% (1+), 25–50% (2+), or >50% (3+) in the cortex	0–3
Total		0–12

**Table 2:** Renal domain of 2019-EULAR/ACR classification criteria for SLE.

Renal Domain	Score
Proteinuria > 0.5 g/24 h	4
Renal biopsy class II or V LN	8
Renal biopsy class III or IV LN	10

**Table 3:** Examples of glucocorticoid dosing and tapering regimens in the initial treatment of LN. Adapted from [92].

	Standard-Dose	Moderate-Dose	Reduced-Dose
	Intravenous methylprednisolone pulses		
	Oral prednisone equivalent (daily)		
Week 0–2	0.8–1 mg/kg (max 80 mg)	0.6–0.7 mg/kg (max 50 mg)	0.5–0.6 mg/kg (max 40 mg)
Week 3–4	0.6–0.7 mg/kg	0.5–0.6 mg/kg	0.3–0.4 mg/kg
Week 5–6	30 mg	20 mg	15 mg
Week 7–8	25 mg	15 mg	10 mg
Week 9–10	20 mg	12.5 mg	7.5 mg
Week 11–12	15 mg	10 mg	5 mg
Week 13–14	12.5 mg	7.5 mg	2.5 mg
Week 15–16	10 mg	7.5 mg	2.5 mg
Week 17–18	7.5 mg	5 mg	2.5 mg
Week 19–20	7.5 mg	5 mg	2.5 mg
Week 21–24	5 mg	<5 mg	2.5 mg
Week > 25	<5 mg	<5 mg	<2.5 mg

**Table 4:** New potential therapies for lupus nephritis.

Molecule	Target	Status
Voclosporin	Calcineurin inhibitor	Approved for adults
Obinutuzumab	CD20	Phase 3 trial
Ofatumumab	CD20	cSLE case series
Telitacicept	BLyS, APRIL	Phase 3 trial
Ianalumab	BLyS	Phase 3 trial
Anifrolumab	IFN-I	Phase 3 trial
Sifalimumab	IFN-α	Phase 2 trial (adult SLE)
Iberdomide	Ikarios, Aiolos	Phase 2 trial (adult SLE)
CAR-T	CD19	cSLE case series
Ravulizumab	C5 complement	Phase 2 trial
Vemircopan	Complement factor D	Phase 2 trial
Secukinumab	IL-17A	Phase 3 trial
Vunakizumab	IL-17A	Phase 2 trial
Guselkumab	IL-23	Phase 2 trial
Daratumumab	CD38	Phase 2 trial
Baricitinib	JAK	Phase 3 trial
Efgartigimod	FcRn	Phase 2 trial
Zetomipzomib	Immunoproteasome	Phase 1/2 trial
Zanubrutinib	BTK	Phase 2 trial
Iscalimab	CD40	Phase 2 trial
Itolizumab	CD6	Phase 1 study

## Data Availability

Not applicable.
